# Intraoperative free margins assessment of oropharyngeal squamous cell carcinoma with confocal laser endomicroscopy: a pilot study

**DOI:** 10.1007/s00405-021-06659-y

**Published:** 2021-02-13

**Authors:** Matti Sievert, Florian Stelzle, Marc Aubreville, Sarina K. Mueller, Markus Eckstein, Nicolai Oetter, Andreas Maier, Konstantinos Mantsopoulos, Heinrich Iro, Miguel Goncalves

**Affiliations:** 1 Department of Otorhinolaryngology, Head and Neck Surgery, University Hospital, University of Erlangen–Nuremberg, Germany, Waldstrasse 1, 91054 Erlangen, Germany; 2 Department of Maxillofacial Surgery, University Hospital, Friedrich-Alexander-Universität Erlangen–Nürnberg, Erlangen, Germany; 3 Institute of Image Understanding and Medical Application of Artificial Intelligence, Technische Hochschule, Ingolstadt, Germany; 4 Institute of Pathology, University Hospital, Friedrich-Alexander-Universität Erlangen–Nürnberg, Erlangen, Germany; 5 Pattern Recognition Laboratory, Computer Science, Friedrich-Alexander-Universität Erlangen–Nürnberg, Erlangen, Germany

**Keywords:** Confocal laser endomicroscopy, Oropharyngeal squamous cell carcinoma, Safe surgical margins, Head and neck malignancies

## Abstract

**Purpose:**

This pilot study aimed to assess the feasibility of intraoperative assessment of safe margins with confocal laser endomicroscopy (CLE) during oropharyngeal squamous cell carcinoma (OPSCC) surgery.

**Methods:**

We included five consecutive patients confirmed OPSCC and planned tumor resection in September and October 2020. Healthy appearing mucosa in the marginal zone, and the tumor margin, were examined with CLE and biopsy during tumor resection. A total of 12,809 CLE frames were correlated with the gold standard of hematoxylin and eosin staining. Three head and neck surgeons and one pathologist were asked to identify carcinoma in a sample of 169 representative images, blinded to the histological results.

**Results:**

Healthy mucosa showed epithelium with uniform size and shape with distinct cytoplasmic membranes and regular vessel architecture. CLE optical biopsy of OPSCC demonstrated a disorganized arrangement of variable cellular morphology. We calculated an accuracy, sensitivity, specificity, PPV, and NPV of 86%, 90%, 79%, 88%, and 82%, respectively, with inter-rater reliability and *κ*-value of 0.60.

**Conclusion:**

CLE can be easily integrated into the intraoperative setting, generate real-time, in-vivo microscopic images of the oropharynx for evaluation and demarcation of cancer. It can eventually contribute to a less radical approach by enabling a more precise evaluation of the cancer margin.

## Introduction

The incidence of oropharyngeal squamous cell carcinoma (OPSCC) is rising in the last decades due to an increased number of infections with oncogenic human papillomavirus [1] and is currently one of the most frequent malignancies in the head and neck region [2]. In early-stage OPSCC, therapeutic options recommend surgical tumor removal or radiotherapy as a single treatment modality [3]. Treatment recommendations for advanced stage OPSCC suggest either primary chemoradiation therapy or primary surgery followed by risk-adapted adjuvant treatment. Both primary radiation and primary surgery proved to be therapeutic options [4].

According to the World Health Organization, squamous cell carcinoma (SCC) is classified into highly differentiated (grade 1), moderately differentiated (grade 2), and poorly differentiated (grade 3) cancer [5]. Grade 1 SCC exhibits a growth pattern that resembles a normal epithelium, whereas grade 3 SCC shows more considerable cell pleomorphism, minimal keratinization, or none at all, high nuclear to cytoplasmic ratio, larger immature cells, and a markedly stromal reaction [5]. Several optical imaging methods, such as narrow-band imaging, fluorescence endoscopy, or optical coherence tomography, have been suggested as having the potential to improve the analysis of mucosal lesion with white light [6–9]. A further imaging modality for visual identification of suspicious mucosal lesions is CLE, magnifying power up to 1000 times using fluorescein to outline the intercellular spaces [9]. While a few studies in the last years analyzed CLE accuracy in detecting malignant mucosal alterations focusing on the diagnosis of a primary lesion, there is no data regarding the assessment of safe margins during oncological surgery [10–13]. CLE provides “real-time” optical biopsies [14], which defines the appropriate resection margin during surgical tumor removal due to its property making cellular structures visible.

This study aimed to assess the diagnostic value of probe-based CLE in identifying the oropharyngeal region's malignant lesions compared to the accepted gold standard of histopathological examination. Additionally, we aimed to assess the feasibility of intraoperative assessment of safe margins with CLE during planned oropharyngeal tumor resection.

## Materials and methods

### Study design

We conducted this prospective pilot study at a tertiary hospital and academic cancer center. The study was approved by the local institutional ethics committee and carried out following the Declaration of Helsinki. We obtained written, informed consent from all study participants.

### Eligibility criteria

A total of five patients with confirmed OPSCC and planed tumor resection were included in this study in September and October 2020. We confirmed malignancy by biopsy out of the center of the tumor in a preceding panendoscopy. Exclusion criteria were a prior head and neck cancer, presence of distant metastasis, pregnancy, thyroid dysfunction, severe kidney failure, and allergy to Fluorescein.

### Technical details

We performed intraoperative imaging using a GastroFlex probe and a 488 nm Cellvizio laser scanning system (Mauna Technologies, Paris, France). The probe has a diameter of 2.6 mm, a penetration depth of 55—65 μm, a field of view of 240 μm, and a resolution of 1 μm. We used 5 ml Fluorescein Alcon 10% (Alcon Pharma, Freiburg, Germany) as an optical imaging dye. Fluorescein distributes to the intercellular spaces and cytoplasmatic components via microcirculation, without staining the cell nuclei. Thus, enabling outline visualization and structural analysis of cellular tissue and the loss of nuclear polarity and abnormal microvasculature patterns which are usually present in tumor tissue [15, 16]. With this imaging method, laser light is emitted and applied to tissue at a selected depth (e.g., 60 µm). The reflected fluorescence of light is refocused for detection. A pinhole excludes scattered or reflected light from other depth planes, thus enabling an increased spatial resolution [17].

### Surgical procedure and intraoperative imaging

**Fig. 1:**
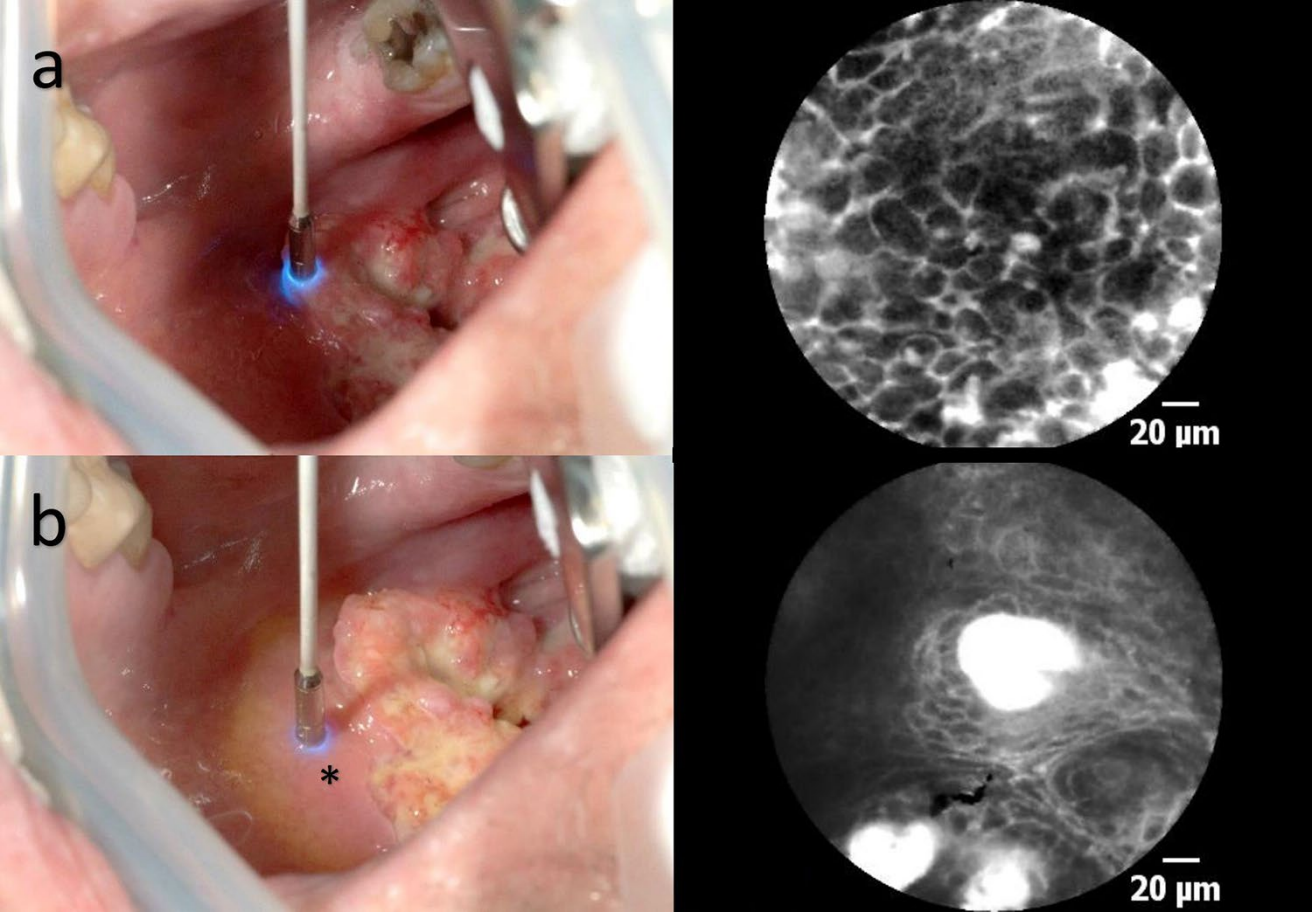
**a** Laser scanning of the tumor. **b** Laser scanning of the macroscopically inconspicuous margin area in the oropharynx (*transition zone, 1–5 mm away from the tumor)

We used an Mc Ivor tongue retractor or a Jennings mouth gag and exposed the tumor. Subsequently, 2.5 ml Fluorescein Alcon 10% was injected intravenously, and after 2 min, the laser scanning unit was initiated (Fig. 1). After around 5 min of examination, we administrated additional 2.5 ml because the quality of imaging began to degrade this time interval [18]. We correlated the CLE imaging with histopathology's gold standard. The regions recorded were marked with suture, or a separate biopsy was performed. The histopathological assessment followed a standard protocol with hematoxylin and eosin (H&E) staining. After completing the CLE examination, we performed the tumor resection with a macroscopic safety margin of 1 cm. Our and international treatment standards were not altered or influenced in any way by the application of CLE.

### Data analysis

The investigator edited CLE data postoperatively using Cellvizio Viewer software 1.6.2. A total of 12.809 CLE frames were analyzed, and 169 representative images from different mucosal areas of the tumor margin, the marginal zone, and the resection margin were selected. All images were of high quality and free of artifacts. Besides, each image contains a corresponding H&E pattern as a reference standard. The 169 representative images were extracted and presented to four medical professionals (blinded examiners) for assessment. The professionals consisted of three head and neck surgeons with confocal microscopy experience and one pathologist without previous experience in this technique. The blinded examiners had to allocate the CLE image into the categories of “healthy” and “carcinoma in situ, or invasive carcinoma.” As has already been mentioned, the maximum penetration depth is approximately 60 µm. Therefore, it is impossible to distinguish carcinoma in situ from invasive carcinoma since stromal invasion cannot be used as a criterion to differentiate between the two [12, 19].

### Statistical analysis

We performed statistical analysis using SPSS version 22.0 (IBM SPSS Statistics for Windows, Version 22.0. Armonk, NY, USA). The sensitivity, specificity, positive predictive value (PPV), negative predictive value (NPV), and accuracy were calculated for each examiner. Assessment of the results was blinded to histological findings. The histological findings were regarded as the reference standard. The inter-rater reliability/agreement was tested statistically using Cohen’s kappa (Cohen’s kappa coefficient) and Fleiss kappa. The *κ*-values were interpreted according to the widely accepted Landis and Koch classification [20]. If the agreement obtained between the two raters is mathematically equivalent to chance, *κ* is zero (no agreement). Values of *κ* between 0 and 0.20 are defined as low, between 0.21 and 0.40 as adequate, between 0.41 and 0.60 as moderate, between 0.61 and 0.80 as substantial, and between 0.81 and 1.0 almost perfect.

## Results

### Patient characteristics

In 08—10/2020, we enrolled five patients (all male; mean age 58.2 years, range 51–61 years) to undergo pharyngeal in vivo CLE during planned transoral tumor resection. Patient characteristics, including stage, are shown in Table 1. All patients underwent transoral tumor resection surgery. One case was performed without the need for reconstruction. The other four cases underwent free flap reconstruction (two with anterior lateral thigh flap and two with radial forearm flap). SCC and healthy mucosa (safe surgical margins) were found in CLE and confirmed by histopathology in all patients.

**Table 1:** Tumor characteristics

Case	Age (years)	Tumor stage	Location		Grade	p16	CLE frames	Time
1	57	pT2	Tonsil	> 0,5 cm	G2	Neg	2217	4 m 37 s
2	61	pT2	Soft palate	> 0,5 cm	N/A	Pos	1960	4 m 5 s
3	51	pT3	Soft palate	> 0,5 cm	G3	Neg	2418	5 m 2 s
4	61	pT2	Soft palate	> 0,5 cm	G3	Neg	3498	7 m 17 s
5	61	pT3	Tonsil	> 0,5 cm	G3	Neg	2716	5 m 40 s

*N/A* not applicable

### CLE image acquisition

The average image acquisition time for each case was 5 min, 20 s (range: 4 min 5 s–7 min 17 s). In amount, we acquired 12,809 CLE frames, which were assessed and classified according to SCC or healthy epithelium.

**Fig. 2:**
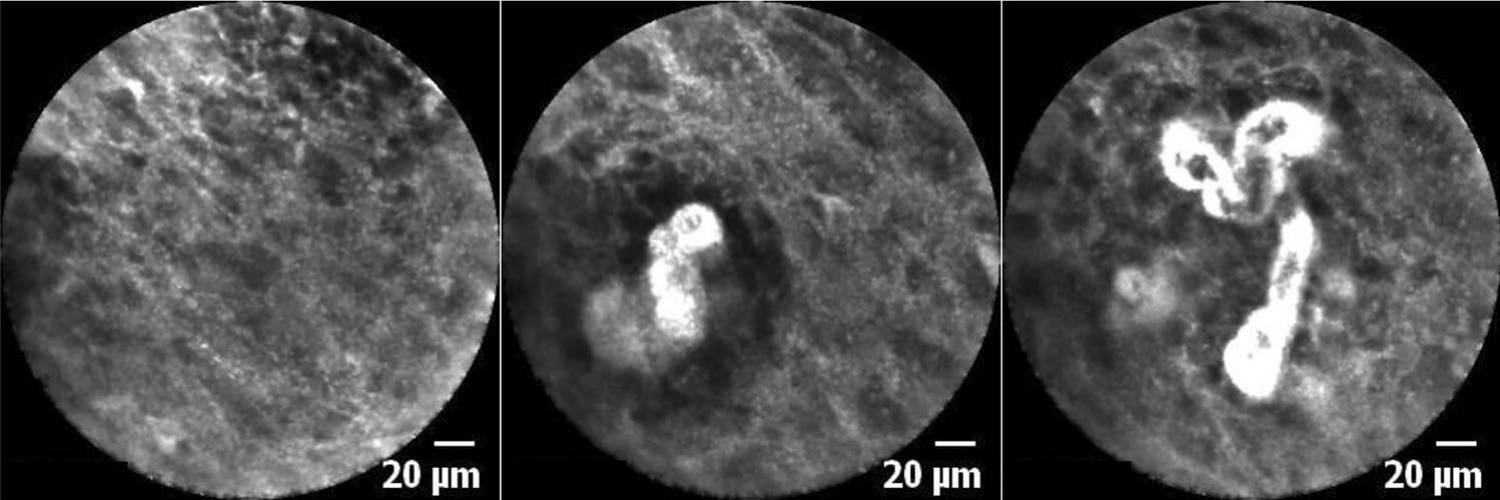
True-positive finding. All raters identified these images as corresponding to SCC. Note the poorly defined intercellular gaps between the cells' irregularity and the intensified fluorescein leakage from the dilated capillaries. All images were taken in the transition zone on macroscopically inconspicuous mucosa

In CLE, SCC is characterized by unorganized tissue architecture whereby the intercellular gaps are not definable in some places. Cells are irregular in shape, the intensity of contrast, and size, with intensified fluorescein leakage from the dilated capillaries (Fig. 2).

**Fig. 3:**
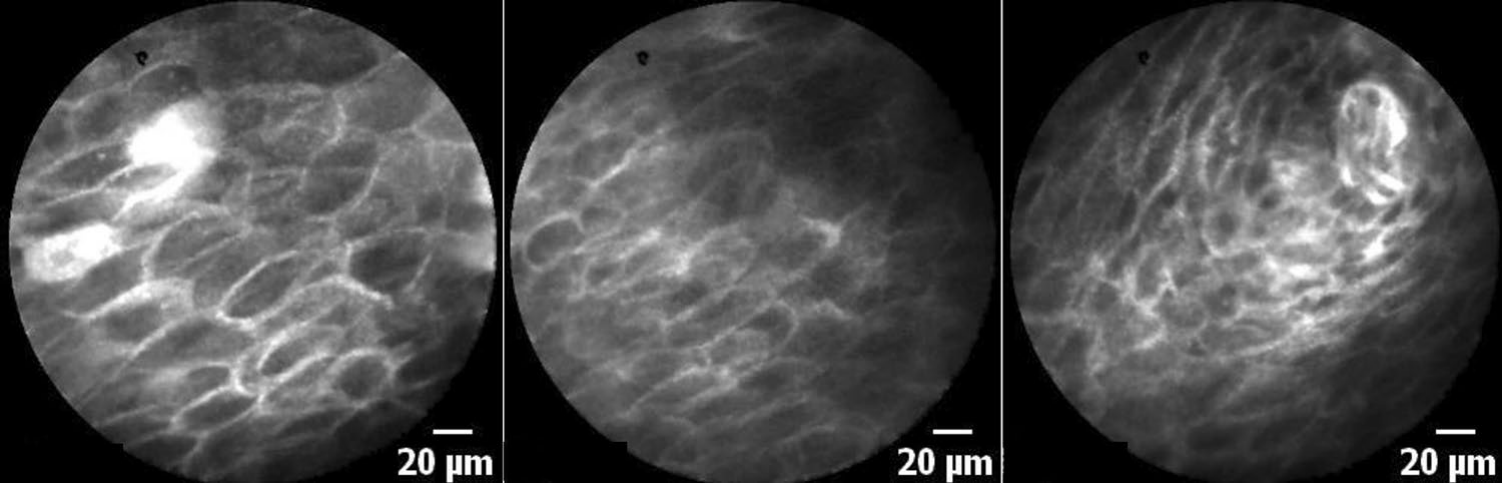
True-negative finding. All raters identified this image as corresponding to healthy epithelium—Note the regularity of the cell borders in a polygonal honeycomb-like pattern. All images were taken in the transition zone

Benign squamous epithelium presents with homogeneous cells of uniform size, shape with distinct cytoplasmic membranes, and regular vessel architecture (Fig. 3). A regular cell border and normal capillaries are the most distinct features in comparison to SCC.

### Diagnostic metrics

The performance of our binary classification is illustrated in Table 2. The examiners achieved an accuracy, sensitivity, specificity, PPV, and NPV were in the range of 79–92%, 84–95%, 52–95%, 77–97%, and 76–86%. In summary, we could show an accuracy, sensitivity, specificity, PPV and NPV of 85.8%, 89.9%, 78.6%, 87.9% and 81.9%, respectively. The four observers' overall agreement was calculated with a Fleiss’ κ of 0.60 for the 169 images and can, therefore, be considered sufficient. In computing the inter-rater reliability in the selective analysis between two examiners, Cohens’ *κ* ranged from 0.32 to 0.78 with a percentage agreement level ranging from 69.2 to 89.3% (Table 3).

**Table 2:** Data analysis by the several reviewers

Diagnostic Metrics	P	S1	S2	S3	All
	Value (95%CI)	Value (95%CI)	Value (95%CI)	Value (95%CI)	Value (95%CI)
Sensitivity	89.72% (82.35–94.76)	95.33% (89.43–98.47)	90.65% (83.48–95.43)	84.11% (75.79–90.46)	89.95% (86.71–92.63)
Specificity	80.65% (68.63–89.58)	51.61% (38.56–64.50)	95.16% (86.50–98.99)	87.10% (76.15–94.26)	78.63% (73.00–83.56)
PPV	88.89% (82.74–93.03)	77.27% (72.38–81.52)	97.00% (91.46–98.99)	91.84% (85.43–95.57)	87.90% (85.10–90.24)
NPV	81.97% (71.93–88.96)	86.49% (72.46–93.97)	85.51% (76.53–91.43)	76.06% (67.03–83.23)	81.93% (77.22–85.85)
Accuracy	86.39% (80.28–91.17)	79.29% (72.39–85.13)	92.31% (87.21–95.84)	85.21% (78.94–90.19)	85.80% (82.94–88.34)

*S* surgeon, *P* pathologist, *PPV* positive predictive value, *NPV* negative predictive value; (95% Confidence Interval)

**Table 3:** Interobserver agreement

Observer pair	Cohens Kappa/Fleiss-Kappa	Agreement (%)
S1 vs S2	0.45	75.2
S1 vs S3	0.32	69.2
S1 vs P	0.44	76.3
S2 vs S3	0.73	86.9
S2 vs P	0.78	89.3
S3 vs P	0.60	81.1
S1 vs S2 vs S3 vs P	0.60	

Interobserver agreement for observer pairs (Cohens Kappa) and for multiple Raters (Fleiss Kappa) as well as percentual agreement

## Discussion

In this pilot study, we report on the feasibility of in-vivo real-time intraoperative assessment of safe margins during tumor resection of OPSCC via confocal laser imaging. The blinded examination of representative images of oropharyngeal cancer and healthy epithelium cells showed a sensitivity and specificity of, respectively, 89.9% and 78.6%. The tumor's exposure was performed in a standardized fashion, and the examination added around 10 min to standard operation time without any side effects. The 2.6 mm CLE probe provided sufficient spatial resolution and tissue contrast to distinguish cellular architecture, borders, and size to characterize healthy and malignant tissue in vivo specimens. Imaging of healthy areas of the pharynx (confirmed by histopathological assessment) after tumor resection allowed for the benign squamous epithelium classification as having cells of uniform size and shape with distinct cytoplasmic membranes and regular vessel architecture. We could also detect a transition between healthy appearing tissue and suspicious lesions observed with CLE, showing that CLE can precisely evaluate the cancer margin. CLE optical biopsy of SCC demonstrated a disorganized and variable cellular morphology, lack of cytoplasmic membrane, which could correlate with H&E staining. The blinded examiners (S1-3 and P) showed a moderate interrater agreement. Available reports on in vivo confocal laser endomicroscopy images on the head and neck are mostly related to the larynx and oral mucosal lesions. Only very few data on oropharyngeal cancer and the tonsillar region is available [10, 12, 13, 21, 22]. Linxweiler et al. examined, among other areas, the tonsillar region of 12 patients. However, this was performed on formalin-fixed samples with topical use of acriflavine or using the cells’ autofluorescence, which does not adequately portray the in vivo intraoperative situation, limiting its transferability [11].

**Fig. 4:**
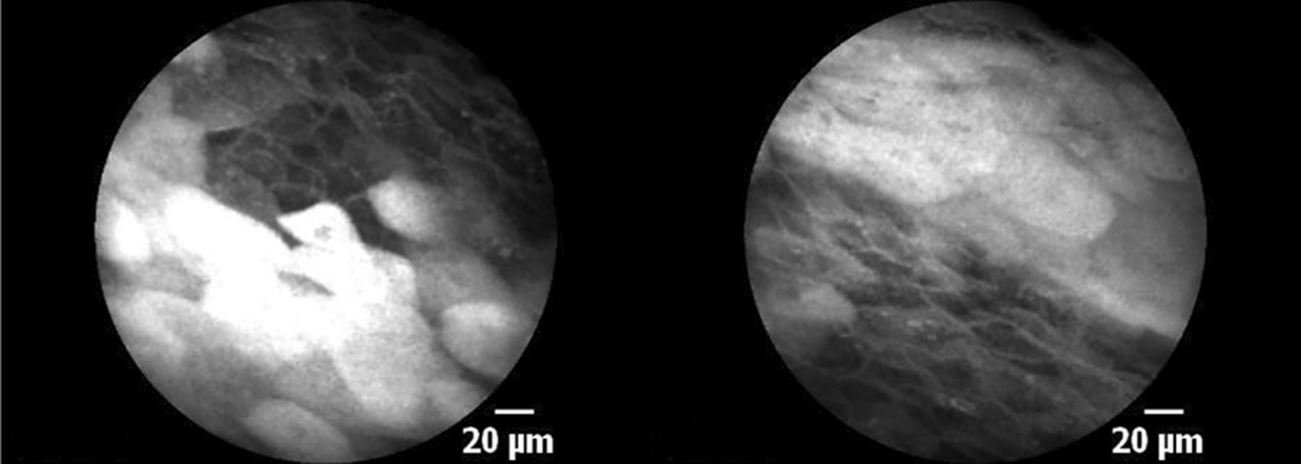
Mucosal mucinous cells. Note the large white cells on CLE. Both images were classified as suspicious by 50% of the rater

We used intravenous fluorescein as a dye to characterize the morphological features of normal epithelium and SCC with CLE. A sufficient resolution of in vivo cellular architecture within a few minutes of fluorescein administration was feasible. CLE image interpretation has a moderate learning curve, and regarding the interrater reliability, there is inconsistency in the literature [13, 21, 23]. Oetter et al. developed and validated a classification and scoring system to diagnose oral SCC through CLE [10]. However, this system has not yet been validated for OPSCC. Indeed, CLE’s application by one of the raters, a maxillofacial surgeon with extensive experience in the surgical treatment and intraoperative use of CLE in oral cancer, shows a relatively low specificity and high sensitivity (S1: 51.6%, 95.3%; Table 2). We postulate that this is due to the atypical aspect of oropharyngeal and tonsillar epithelium compared to the oral mucosa. Large mucosal cells are typical in the oropharyngeal epithelium and were not considered in the DOI Classification by Oetter et al. (Fig. 4).

An accurate histopathological diagnosis of OPSCC is vital in guiding clinical management. CLE images have a similar resolution to traditional H&E and thus could serve as valuable adjunct technology by potentially minimizing the need for intraoperative frozen section analysis. Pathological assessment of frozen sections and biopsy specimens does not typically involve the surgeon; however, a trained head and neck surgeon can perform CLE image interpretation for a real-time distinction of normal from abnormal tissue. The ability to sample the entirety of a tumor and surrounding tissue at the time of surgery may help guide the procedure's extension. This is of paramount relevance since an extensive resection may require free flap reconstructive surgery with considerable morbidity and prolonged operating time. Current evidence suggests that a safe margin of 1 mm may be sufficient for OPSCC [24]. However, intraoperative frozen sections are limited by the under-sampling, processing time, and the use of the tissue of interest [25], thus underscoring the need for improved intraoperative diagnostics. CLE enables a rapid scanning of the tumor margin and could potentially contribute to a more accurate resection, thus reducing the resection extension. Although this technique’s potential has been recognized over the years by a few groups, it has not yet achieved a broad clinical application.

A further possibility to objectivize CLE findings is utilizing automatic classification [26]. Aubreville et al. showed that an approach based on transfer learning from intermediate endpoints within a pre-trained inception v3 network with preprocessing could reach an overall 94.8% accuracy, significantly improving overall performance over the traditional state of the art feature-based machine learning approaches [26]. Automatic classification methods for confocal laser endomicroscopy in the head and neck were developed for vocal cords and oral mucosa, but until this point, there is no available data for the oropharyngeal region, especially for the palatine tonsil. Prerequisite in developing such an automatic classification method based on deep learning-based approaches in acquiring large amounts of data and the correct labeling of such images in the healthy epithelium of a specific anatomic region and cancerous cells. This study demonstrated image acquisition's feasibility in the oropharyngeal region and CLE application in an intraoperative setting. We showed that a blinded examination of these images could be performed with 85.8% accuracy-based solely on known characteristics of healthy epithelium and cancerous tumor cells in general. Interestingly, compared to a previous study on the transferability of automatic classification based on algorithms trained with images of vocal cords applied on the oral cavity and vice versa, an accuracy of 68.5% and 89.5% was found. This suggests that the epithelium of these areas and the carcinomas that arise are similar on CLE; however, there seem to be significant differences that limit its detection rate. The same difficulties can also be pointed to the blinded examiners, mostly in the oral cavity (S1) and vocal cords/Larynx (S2, S3).

The present work increases the information and knowledge until now less examined anatomic region. The absence of classification criteria and small data available limit CLE's clinical application in head and neck until this day. CLE is also associated with considerable costs as a scanning unit costs around $200 000, and a single application costs $275 ($250 for one probe use and $25 for the contrast agent Fluorescein 10% 5 ml) with a significant decrease in image quality after 8 min of recording [17]. Since CLE was not yet demonstrated sufficient clinical added value, the costs are difficult to justify in the face of increased economic pressure outside of clinical trials. CLE has a considerable potential to aid intraoperative resection, dependent on further validation in clinical trials.

## Conclusion

CLE is a promising imaging technology that may aid in intraoperative decision-making during oropharyngeal tumor removal CLE can be used to generate real-time, in-vivo microscopic examination of the oropharynx for evaluation and demarcation of cancer. This evaluation can be easily integrated into the intraoperative setting to help the surgeon guide its resection and eventually contribute to a less radical approach.
